# The effects of air pollution on mortality and clinicopathological features of esophageal cancer

**DOI:** 10.18632/oncotarget.17266

**Published:** 2017-04-20

**Authors:** Xiaochen Huang, Shanghui Guan, Jiangfeng Wang, Linli Zhao, Yibin Jia, Zilong Lu, Cuiping Yin, Shengsi Yang, Qingxu Song, Lihui Han, Cong Wang, Jingyi Li, Wei Zhou, Xiaolei Guo, Yufeng Cheng

**Affiliations:** ^1^ Department of Radiation Oncology, Qilu Hospital of Shandong University, Jinan 250012, Shandong, China; ^2^ Department of Noncommunicable Disease Control and Prevention, Shandong Center for Disease Control and Prevention, Jinan 250014, Shandong, China; ^3^ Department of Rehabilitation, Qilu Hospital of Shandong University, Jinan 250012, Shandong, China

**Keywords:** air pollution, PM_10_, esophageal cancer, mortality, clinicopathological features

## Abstract

This study aimed to estimate the associations between air pollution and esophageal cancer. In the ecologic cross-sectional study, correlation analyses were made between city-level mean concentrations of particulate matter less than 10μm in aerodynamic diameter (PM_10_), SO_2_, NO_2_ and city-level age-standardized mortality rates of esophageal cancer in Shandong Province, China. PM_10_ (*p*=0.046) and NO_2_ (*p*=0.03) both had significant linear correlations with esophageal cancer mortality rates. After introducing smoking as a risk factor in models of multiple linear regression analyses, PM_10_ was still an independent risk factor that increased esophageal cancer mortality rates. This study further compared clinicopathological features of 1,255 eligible esophageal squamous cell carcinoma patients by dividing them into different pollution level groups. There was statistically significant difference in gender distributions (*p*=0.02) between groups after subgroup analysis. Female patients accounted for a higher proportion in the high PM_10_ level group than in the low PM_10_ level group. It suggested that females were more sensitive to higher PM_10_ level pollution. The features that manifested the degree of malignancy of esophageal cancer, including primary tumor invasion, regional lymph nodes metastasis, histological grade, stage, lymph-vascular invasion and tumor size demonstrated no statistically significant difference between groups.

## INTRODUCTION

The International Agency for Research on Cancer (IARC) Working Group classified outdoor air pollution and particulate matter in outdoor air pollution as carcinogenic to humans (Group 1) [1]. Particulate matter is a mixture of extremely small particles and liquid droplets absorbing organic chemicals, acids and metals that vary in size, composition, and origin [2]. The long-term effects of air pollution on lung cancer have been consistently documented worldwide. The Harvard Six Cities study and the American Cancer Society study were two landmark prospective cohort studies in this area. Results suggested that air pollution, especially particulate air pollution, increased lung cancer mortality [2, 3]. However, the effects of air pollution on esophageal cancer appear to be less clear.

Esophageal cancer is one of the most commonly diagnosed cancers in China [4]. Interestingly, there exist striking geographical variations in the incidence and mortality rates of esophageal cancer, which implies that environmental factors might play a major role in cancer development [5]. Esophageal cancer shares some epidemiological features with lung cancer. However, to our knowledge, no prior studies have ever existed in China estimating the associations between air pollution and esophageal cancer. Thus, we assumed that the already proved risk factor for lung cancer, namely, air pollution, might also play a significant role in esophageal cancer. We proposed three hypotheses: (1) Air pollution might contribute to excess mortality of esophageal cancer. (2) Air pollution might increase the degree of malignancy in esophageal cancer patients. (3) Females living in the more polluted areas might be more vulnerable. The objectives of our study were to estimate the impact of air pollution, including particulate matter less than 10μm in aerodynamic diameter (PM_10_), SO_2_ and NO_2_ on the mortality rates of esophageal cancer in Shandong Provence, China, and the effect of PM_10_ on the clinicopathological features of esophageal squamous cell carcinoma (ESCC) of the patients in Qilu Hospital, Shandong, China.

## RESULTS

### Ecologic cross-sectional study

**Figure 1:**
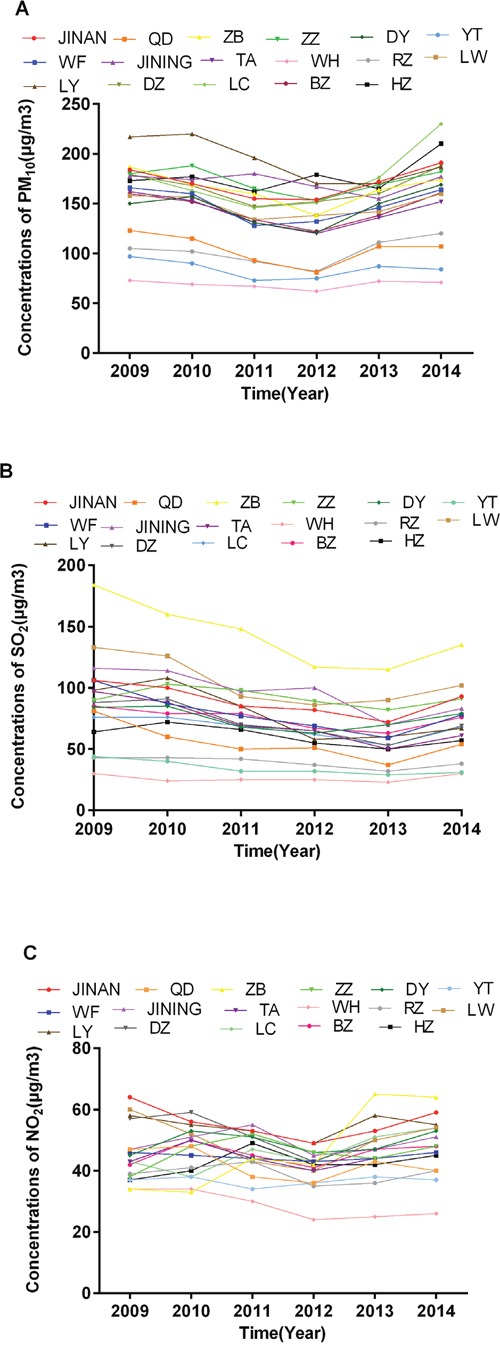
Annual mean concentrations of PM_10_, SO_2_ and NO_2_ during 2009-2014 of all 17 cities in Shandong Province Each color represented a certain city. **(A)** showed annual mean concentrations of PM_10_ (μg/m^3^); **(B)** showed annual mean concentrations of SO_2_ (μg/m^3^); **(C)** showed annual mean concentrations of NO_2_ (μg/m^3^). JINAN: Jinan; QD: Qingdao; ZB: Zibo; ZZ: Zaozhuang; DY: Dongying; YT: Yantai; WF: Weifang; JINING: Jining; TA: Tai’an; WH: Weihai; RZ: Rizhao; LW: Laiwu; LY: Linyi; DZ: Dezhou; LC: Liaocheng; BZ: Binzhou; HZ: Heze.

**Figure 2:**
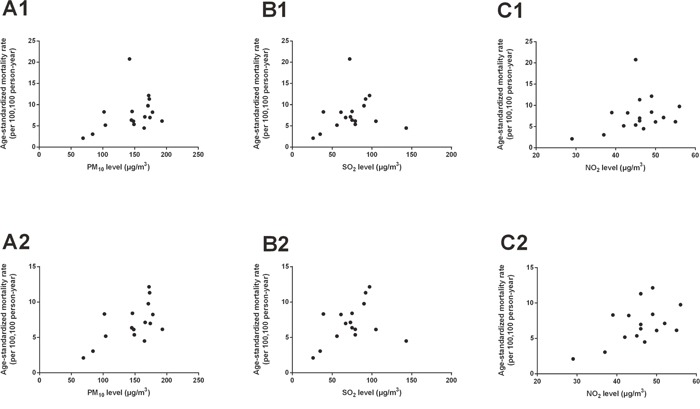
Scatter plots of air pollution concentrations against esophageal cancer mortality rates in Shandong Province The mean concentrations of PM_10_, SO_2_, NO_2_ were plotted against the age-standardized esophageal cancer mortality rates of the year 2015, respectively. **(A1)**, **(B1)** and **(C1)** showed scatter plots including Tai’an City; **(A2)**, **(B2)** and **(C2)** showed scatter plots excluding Tai’an City.

The city-level annual mean concentrations of PM_10_, SO_2_ and NO_2_ during 2009-2014 were demonstrated in Figure 1. The detailed data were listed in Supplementary Table 1. The interannual variations were not pronounced, so we used mean pollution data during the observed period to represent the pollution level of a certain city. Figure 2 showed the scatter plots of air pollution against esophageal cancer mortality rates. The city-level mean concentrations of PM_10_, SO_2_, NO_2_ were plotted against the city-level age-standardized mortality rates of esophageal cancer in 2015, respectively. Figure 2 (A1), (B1) and (C1) showed 17 points each representing a certain city in this study. As shown in the scatter plots, there was a point with extraordinary high mortality rate in each plot. That point represented the Tai’an City. Tai’an has been a high risk area of esophageal cancer in China for decades. The specific carcinogenic reasons were not clear yet. But they might only reflect regional situations and cannot be applied extensively. After excluding the data of Tai’an, the correlations between air pollution and esophageal cancer mortality rates became stronger, as demonstrated in Figure 2 (A2), (B2) and (C2). Table 1 listed *r/r_s_* values and *p* values of correlation analyses. Results including and excluding Tai’an City were listed separately. PM_10_ levels and NO_2_ levels both had significant linear correlations with age-standardized esophageal cancer mortality rates. Table 2 showed only statistically significant models of multiple linear regression analyses. Collinearity diagnostics was made on each model and there was no collinearity between the independent variables. After introducing smoking rates as an adjustment, PM_10_ was still an independent risk factor that increased esophageal cancer mortality rates in Shandong Province. The entire models of multiple linear regression analyses were listed in Supplementary Table 2. Supplementary Figure 1 and Supplementary Table 3 demonstrated the scatter plots and results of correlation analyses between air pollution and mortality rates of all cancers combined, diabetes mellitus and ischemic heart disease. There was no statistically significant linear correlation. The results further proved the specific effect of air pollution on esophageal cancer rather than a general effect.

**Table 1:** Correlation analyses between air pollution and esophageal cancer mortality rates

Correlation analysis	*r/r_s_*	*p*
Including Tai’an City		
PM_10_ & esophageal cancer mortality rates	0.38 ^a^	0.14 ^a^
SO_2_ & esophageal cancer mortality rates	0.26 ^a^	0.32 ^a^
NO_2_ & esophageal cancer mortality rates	0.36 ^a^	0.16 ^a^
Excluding Tai’an City		
PM_10_ & esophageal cancer mortality rates	0.51 ^a^	**0.046** ^a^
SO_2_ & esophageal cancer mortality rates	0.33	0.21
NO_2_ & esophageal cancer mortality rates	0.53	**0.03**

Correlation analyses were processed between city-level mean concentrations of PM_10_, SO_2_, NO_2_ and city-level age-standardized mortality rates of esophageal cancer in 2015, respectively. The results of *r/r_s_* values and *p* values were listed. Normality test was made for each variable. Pearson correlation analysis was used when both variables followed normal distributions. Spearman rank correlation analysis was used when either variable was non-normally distributed. *p* values less than 0.05 were considered statistically significant.

^a^ Results gained using Spearman rank correlation analysis

**Table 2:** Multiple linear regression modeling of factors associated with esophageal cancer mortality

Model		*β* (95% CI)	Std. error	*t*	*R^2^*	*F*	*p*
1	Constant	4.56 (-2.69, 11.81)	3.29	1.39	0.62	9.14	**<0.01**
	Smoking	-0.25 (-0.53, 0.04)	0.13	-1.89			
	PM_10_	**0.05 (0.03, 0.08)**	0.01	4.18			
2	Constant	5.44 (-1.90, 12.78)	3.29	1.65	0.67	6.90	**0.01**
	Smoking	-0.29 (-0.58, 0.01)	0.13	-2.18			
	PM_10_	**0.07 (0.03, 0.10)**	0.02	4.02			
	SO_2_	-0.03 (-0.07, 0.02)	0.02	-1.24			

Multiple linear regression analyses were run with the combination of mean concentrations of PM_10_, SO_2_, NO_2_ and city-level smoking rates. Esophageal cancer mortality rates were considered as dependent variable. Mean concentrations of PM_10_, SO_2_, NO_2_ and city-level smoking rates were considered as independent variables. Collinearity diagnostics was made on each model and there was no collinearity between the independent variables. Statistically significant multiple linear regression analyses models were listed. Bonferroni correction was applied. *p* values less than 0.05/n were considered statistically significant, where n was the number of independent variables.

### Clinicopathological features between different groups

Table 3 summarized baseline characteristics of the 1,255 study participants in Qilu Hospital. As presented in Table 3, the sex ratio (male versus female) was 4.09 in the high PM_10_ level group and 6.38 in the low PM_10_ level group. Though there were more male patients than female patients in both groups, the proportion of female patients elevated in high PM_10_ level group. 60.32% participants had T3 or T4 staging of the primary tumor invasion, 44.62% participants had regional lymph nodes metastasis, and 83.11% participants had histological grade of 2 or 3. These resulted in the relatively advanced clinical stages for most patients (stage II and stage III accounted for 87.49%) in this study. Table 4 presented the comparison of clinicopathological features between groups. Cigarette-smoking status was introduced as a confounding factor to adjust the bias. There was statistically significant difference in gender distributions (*p*=0.02) between high PM_10_ level group and low PM_10_ level group after subgroup analysis. In the never-smokers subgroup, the effect of smoking has been eliminated. Female patients accounted for a higher proportion in the high PM_10_ level group than the low PM_10_ level group. The sex ratio (male versus female) was 1.20 in high PM_10_ level group and 2.09 in the low PM_10_ level group. The diagnostic age showed no statistically significant difference. The features that manifested the degree of malignancy of esophageal cancer, including primary tumor invasion (T stage), regional lymph nodes metastasis (N stage), histological grade, stage, lymph-vascular invasion and tumor size demonstrated no statistically significant difference between groups.

**Table 3:** Baseline characteristics of study participants

Variables	PM_10_		*χ^2^/t*	*p*
	Low	High		
Gender			5.31	**0.02**
Male	236 (18.80%)	789 (62.87%)		
Female	37 (2.95%)	193 (15.38%)		
Marital status at diagnosis			2.02	0.37
Married	267 (21.27%)	951 (75.78%)		
Unmarried	0	7 (0.56%)		
Widowed	6 (0.48%)	24 (1.91%)		
Age at diagnosis	60.67 ± 8.43	60.55 ± 7.88	0.22	0.83
Employment status			0.36	0.95
Employed	40 (3.19%)	145 (11.55%)		
Self-employed	171 (13.63%)	628 (50.04%)		
Unemployed	16 (1.27%)	50 (3.98%)		
Retired	46 (3.67%)	159 (12.67%)		
Alcohol intake status			6.93	0.07
Never	99 (7.89%)	436 (34.74%)		
Current	157 (12.51%)	503 (40.08%)		
Former	17 (1.35%)	42 (3.35%)		
Cigarette -smoking status			0.68	0.71
Never	108 (8.61%)	415 (33.07%)		
Current	131 (10.44%)	446 (35.54%)		
Former	34 (2.71%)	121 (9.64%)		
Comorbidities			<0.01	0.97
None	203 (16.18%)	729 (58.09%)		
One or more	70 (5.58%)	253 (20.16%)		
Multiple primary malignancies			1.19	0.28
No	260 (20.72%)	949 (75.62%)		
Yes	13 (1.04%)	33 (2.63%)		
Family history of all kinds of cancers			0.11	0.74
No	263 (20.96%)	950 (75.70%)		
Yes	10 (0.80%)	32 (2.55%)		
Year of surgery			8.38	0.14
2010	49 (3.90%)	165 (13.15%)		
2011	53 (4.22%)	171 (13.63%)		
2012	66 (5.26%)	209 (16.65%)		
2013	43 (3.43%)	209 (16.65%)		
2014	61 (4.86%)	228 (18.17%)		
Tumor location			4.05	0.26
Cervical	6 (0.48%)	25 (1.99%)		
Upper thoracic	21 (1.67%)	52 (4.14%)		
Middle thoracic	157 (12.51%)	620 (49.40%)		
Lower thoracic	88 (7.01%)	285 (22.71%)		
Primary tumor invasion			2.16	0.71
Tis	9 (0.72%)	21 (1.67%)		
T1	21 (1.67%)	95 (7.57%)		
T2	75 (5.98%)	273 (21.75%)		
T3	127 (10.12%)	447 (35.62%)		
T4	40 (3.19%)	143 (11.39%)		
Regional lymph nodes metastasis			6.78	0.08
N0	165 (13.15%)	530 (42.23%)		
N1	52 (4.14%)	258 (20.56%)		
N2	43 (3.43%)	139 (11.08%)		
N3	13 (1.04%)	55 (4.38%)		
Distant metastasis			0.24	0.63
M0	272 (21.67%)	980 (78.09%)		
M1	1 (0.08%)	2 (0.16%)		
Histological grade			2.79	0.25
G1	46 (3.67%)	166 (13.23%)		
G2	123 (9.80%)	391 (31.16%)		
G3	104 (8.29%)	425 (33.86%)		
Stage			3.07	0.55
0	9 (0.72%)	20 (1.59%)		
I	23 (1.83%)	98 (7.81%)		
II	129 (10.28%)	435 (34.66%)		
III	110 (8.76%)	424 (33.78%)		
IV	1 (0.08%)	2 (0.16%)		
Lymph-vascular invasion			0.26	0.61
Yes	9 (0.72%)	39 (3.11%)		
No	264 (21.04%)	943 (75.14%)		
Tumor size (cm)	3.87 ± 2.00	3.86 ± 1.70	0.08	0.94

**Table 4:** Clinicopathological features between different groups

PM_10_	Cigarette -smoking status						*χ^2^/F*	*p*
	Never		Current		Former			
	Low (n = 108)	High (n = 415)	Low (n = 131)	High (n = 446)	Low (n = 34)	High (n = 121)		
Tumor location							0.09	0.76
Cervical	5 (0.40%)	16 (1.27%)	1 (0.08%)	7 (0.56%)	0	2 (0.16%)		
Upper thoracic	8 (0.64%)	29 (2.31%)	10 (0.80%)	18 (1.43%)	3 (0.24%)	5 (0.40%)		
Middle thoracic	58 (4.62%)	256 (20.40%)	78 (6.22%)	285 (22.71%)	21 (1.67%)	79 (6.29%)		
Lower thoracic	37 (2.95%)	114 (9.08%)	41 (3.27%)	136 (10.84%)	10 (0.80%)	35 (2.79%)		
Primary tumor invasion							<0.01	0.96
Tis	3 (0.24%)	13 (1.04%)	5 (0.40%)	6 (0.48%)	1 (0.08%)	2 (0.16%)		
T1	11 (0.88%)	47 (3.75%)	8 (0.64%)	37 (2.95%)	2 (0.16%)	11 (0.88%)		
T2	32 (2.55%)	137 (10.92%)	36 (2.87%)	103 (8.21%)	7 (0.56%)	33 (2.63%)		
T3	50 (3.98%)	167 (13.31%)	61 (4.86%)	230 (18.33%)	16 (1.27%)	50 (3.98%)		
T4	12 (0.96%)	50 (3.98%)	20 (1.59%)	68 (5.42%)	8 (0.64%)	25 (1.99%)		
Regional lymph nodes							1.29	0.26
N0	70 (5.58%)	248 (19.76%)	77 (6.14%)	215 (17.13%)	18 (1.43%)	67 (5.34%)		
N1	21 (1.67%)	98 (7.81%)	21 (1.67%)	128 (10.20%)	10 (0.80%)	32 (2.55%)		
N2	13 (1.04%)	46 (3.67%)	27 (2.15%)	74 (5.90%)	3 (0.24%)	19 (1.51%)		
N3	4 (0.32%)	23 (1.83%)	6 (0.48%)	29 (2.31%)	3 (0.24%)	3 (0.24%)		
Histological grade							1.12	0.29
G1	18 (1.43%)	72 (5.74%)	22 (1.75%)	74 (5.90%)	6 (0.48%)	20 (1.59%)		
G2	56 (4.46%)	169 (13.47%)	54 (4.30%)	174 (13.86%)	13 (1.04%)	48 (3.82%)		
G3	34 (2.71%)	174 (13.86%)	55 (4.38%)	198 (15.78%)	15 (1.20%)	53 (4.22%)		
Stage							0.62	0.43
0	3 (0.24%)	12 (0.96%)	5 (0.40%)	6 (0.48%)	1 (0.08%)	2 (0.16%)		
I	12 (0.96%)	47 (3.75%)	9 (0.72%)	40 (3.19%)	2 (0.16%)	11 (0.88%)		
II	57 (4.54%)	205 (16.33%)	60 (4.78%)	179 (14.26%)	12 (0.96%)	51 (4.06%)		
III	36 (2.87%)	148 (11.79%)	55 (4.38%)	219 (17.45%)	19 (1.51%)	57 (4.54%)		
IV	0	2 (0.16%)	1 (0.08%)	0	0	0		
Lymph-vascular invasion							0.31	0.58
No	106 (8.45%)	402 (32.03%)	125 (9.96%)	423 (33.71%)	33 (2.63%)	118 (9.40%)		
Yes	2 (0.16%)	13 (1.04%)	6 (0.48%)	23 (1.83%)	1 (0.08%)	3 (0.24%)		
Tumor size (cm)	3.80 ± 1.72	3.61 ± 1.68	3.75 ± 1.81	4.06 ± 1.68	4.57 ± 3.13	4.00 ± 1.74	<0.01	0.98
Diagnostic age	61.32 ± 9.49	60.92 ± 7.78	60.01 ± 7.52	59.59 ± 7.93	61.12 ± 8.20	62.81 ± 7.57	0.08	0.78
Gender							5.10	**0.02**
Male	73 (5.82%)	226 (18.01%)	129 (10.28%)	443 (35.30%)	34 (2.71%)	120 (9.56%)		
Female	35 (2.79%)	189 (15.06%)	2 (0.16%)	3 (0.24%)	0	1 (0.08%)		
Comorbidities							<0.01	0.99
No	81 (6.45%)	304 (24.22%)	100 (7.97%)	351 (27.97%)	22 (1.75%)	74 (5.90%)		
Yes	27 (2.15%)	111 (8.84%)	31 (2.47%)	95 (7.57%)	12 (0.96%)	47 (3.75%)		

The most relevant confounder, cigarette-smoking status, was introduced as an adjustment to minimize the bias. Cochran-Mantel-Haensel chi-square test was used for categorical variables and analysis of covariance was used for numerical variables. Whole percentages were used. *p* values less than 0.05 were considered statistically significant.

## DISCUSSION

In ecologic cross-sectional study, we observed positive associations between air pollution levels and esophageal cancer mortality rates in Shandong Province. In the correlation analyses, higher PM_10_ levels and NO_2_ levels were associated with increased esophageal cancer mortality. After introducing city-level smoking rates as an adjustment, PM_10_ was still an independent risk factor that contributed to excess esophageal cancer mortality. Although this was only an ecologic cross-sectional study, and link city-level air pollution data, mortality rates and smoking rates together. The results were statistically significant and have never been reported in esophageal cancer before.

As mentioned above, Tai’an City showed extraordinary high esophageal cancer mortality rate. Tai’an has been a high risk area of esophageal cancer in China for decades. The high incidence and mortality rates were recorded back in 1970s [6]. The specific carcinogenic reasons were not clear yet. Possible explanations are listed below. First of all, deoxynivalenol was found in samples of family-stored food crops in Tai’an [7]. Deoxynivalenol is a trichothecene mycotoxin. In ecologic surveys in China, Deoxynivalenol was linked with a higher incidence of esophageal cancer [8, 9]. Secondly, there were three counties with extremely high prevalence of esophageal cancer in Tai’an City, namely Feicheng, Dongping and Ningyang. They are all located along Dawen River. So local experts considered living along the Dawen River as a risk factor for the severe water contamination. However, these findings have not been universally acknowledged. The high esophageal cancer incidence and mortality might only reflect specific regional situations and cannot be applied extensively. So it was reasonable to exclude the data of Tai’an in the ecologic analyses.

In the comparison of clinicopathological features between groups, female patients accounted for a higher proportion in the high PM_10_ level group than the low PM_10_ level group (*p*=0.02). In the never-smokers subgroup (Table 4), the effect of smoking has been eliminated. The sex ratio (male versus female) was 1.20 in high PM_10_ level group and 2.09 in the low PM_10_ level group. It suggested that high PM_10_ concentrations posed a threat to female citizens. There is a striking male predominance in the incidence of esophageal cancer worldwide [4, 10]. Males tend to have bad lifestyle habits such as tobacco use. The population smoking rates in males are much higher than that in females. And smoking is a widely acknowledged risk factor for ESCC [11]. But in the never-smokers subgroup, the effect of smoking has been eliminated and therefore highlighted the carcinogenicity of particulate matter. Unlike the gender difference in the exposure of smoking, PM_10_ is widespread in the ambient air which puts males and females under the same hazardous situation. This might explain the increased proportion of female patients in the high PM_10_ level group.

In contrary to our hypothesis, PM_10_ didn't increase the degree of malignancy of ESCC (Table 4). The possible explanations are as follows. First of all, the atmospheric environment of the entire Shandong Province was more seriously polluted comparing to the observed areas in western counties. In the Harvard Six City study, the annual mean concentration of PM_10_ in the most polluted city was 46.5μg/m^3^ [2]. However, the lowest annual mean concentration of PM_10_ ever recorded was 62.0μg/m^3^ in 2012 in Weihai City. So in this comparison, even the cities of “low PM_10_ level group” were actually heavily polluted. Second, delay in diagnosis and selection bias resulted in relatively advanced stages for most patients in both groups, which might partly weaken the possible influence of PM_10_ on the degree of malignancy of ESCC. What's more, PM_10_ might have little influence on the clinicopathological features of ESCC, or the influence only contributed marginally that could be covered up by other factors. These findings appeared to be negative and meaningless. Nevertheless they might suggest that even the people living in less contaminated areas were actually under insecure circumstances because relatively advanced clinical stages were found among most patients in both groups.

**Figure 3:**
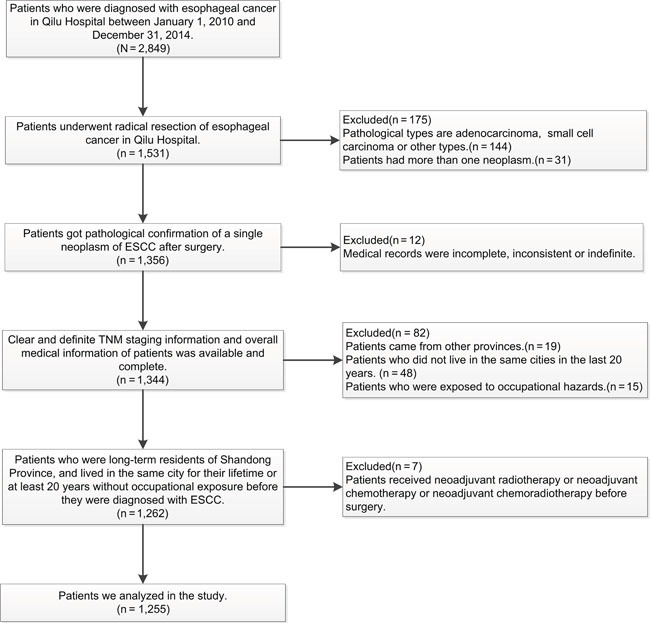
Inclusion criteria and exclusion criteria for patients in the study

Several strengths should be noticed in our study. Most epidemiological and clinical studies focusing on the carcinogenicity of air pollution were done in developed countries where annual mean air pollution levels were much lower than those in China [2, 3, 12]. On the other hand, Iran has both high prevalence of esophageal cancer and severe air pollution. Researchers have done remarkable studies on the relationship of polycyclic aromatic hydrocarbons (PAHs, a major toxic contaminants that are widely dispersed in the environment and highly detected from atmospheric particulate matters [13, 14]) and ESCC [15] in Iran, but they have never linked air pollution with ESCC together. We suspected that lacking of environmental monitoring data could explain the absence of analysis of the obvious phenomenon. However, our study was carried out in a populous province with both severe air pollution problems as well as environmental monitoring data, which provided an opportunity to study the link between air pollution and esophageal cancer. Besides, we followed systematic inclusion and exclusion criteria to screen subjects (Figure 3). For instance, to better minimize the bias caused by lifestyle, eating habits and other factors, we only included long-term residents of Shandong Province. We also excluded patients who were exposed to occupational hazards (coke or gas worker, steel or foundry worker [16], miners [17]), patients who had more than one neoplasm and patients who received neoadjuvant therapies to minimize the bias.

The limitations of this study are listed as follows. First of all, in the ecologic study, there were not enough data points included. In the multiple linear regression analyses, when we excluded the data from Tai’an City and two other cities which lacked smoking registry data, there were only 14 points left. Even though PM_10_ was still an independent risk factor that increased esophageal cancer mortality rates after introducing smoking as an adjustment, the models were not robust enough for the amount of samples. However, the analyses provided us a unique perspective on a provincial level. Secondly, we only gathered the city-level pollution data in limited years. The pollution levels should reflect the cumulative burden of exposure when linked to the development of cancer. Pollution data for an extended period before the study might be more representative. However, the interannual variations of PM_10_, SO_2_ and NO_2_ were not pronounced (Figure 1). So the annual mean pollution data during the limited years could partially reflect exposure to air pollution before the study period. Besides, pollution levels in a certain city were related to population density, energy consumption and meteorological conditions, which were relatively stable for a long time [18]. Therefore we assumed that the mean pollution data during the observed period could have equal representation of the pollution level of a certain city. Thirdly, we used the index PM_10_ instead of particulate matter less than 2.5μm in aerodynamic diameter (PM_2.5_) when the properties of PM_2.5_ might make it a better index to evaluate ambient pollution. But PM_2.5_ wasn't extensively monitored around the province until the beginning of 2016 according to the National Standard of China, Ambient Air Quality Standard (GB3095-2012) [19]. Meanwhile, PM_10_ has been routinely monitored and the data were accessible. In addition, PM_10_ was proved to be mostly comprised of PM_2.5_. PM_2.5_ accounted for 66% and 62% of indoor and outdoor PM_10_, respectively [20]. This made PM_10_ a satisfactory substitute for PM_2.5_ in our study.

Many studies have verified that PAHs were detected in PM_10_ or in the ambient air [13, 14, 18, 21, 22]. PAHs are a large class of organic compounds that are products of incomplete combustion of fossil fuels and other organic materials. They are widespread in the environment [23]. In one study conducted in continental Croatia, nine PAHs were measured in PM_10_ particles. This was one of the most direct evidences that particulate-phase PAHs were absorbed onto PM_10_ particles [14]. Meanwhile PAHs and the most representative PAH, Benzo[a]pyrene, were confirmed as causal roles on the development of esophageal cancer [15, 24, 25]. A number of PAHs were classified as carcinogenic or probably carcinogenic to humans by the IARC Working Group [23]. A case-control study performed in Sweden found an association between exposure to PAHs and ESCC risk, which provided epidemiological evidence [16]. The association was also proved in experimental studies. A very strong dose-response relationship was detected between intensity of esophageal tissue staining with 8E11 antibody (an antibody that raised against a certain PAH) and ESCC risk in Golestan Province, Iran, where the incidence of ESCC was once recorded to be the highest in the world [15]. Benzo[a]pyrene was the most widely studied PAH, and it was well characterized toxicologically. Besides, Benzo[a]pyrene alone contributed more than 50% of the carcinogenic potency of all measured PAHs [14]. Benzo[a]pyrene was defined as group 1 carcinogen to humans by the IARC Working Group [23], and was verified to induce ESCC in experimental animals [24, 25]. Therefore, we tentatively put forward a possible theoretical link that PAHs were detected in PM_10_ or in the ambient air. Meanwhile PAHs were confirmed as causal roles on the development of esophageal cancer. So we might come to the conclusion that air pollution, especially PM_10_, had an adverse effect on esophageal cancer.

Tobacco smoking is carcinogenic to humans (Group 1), and can cause a list of cancers including esophageal cancer [11, 26–29]. PAHs has been detected in tobacco smoke for a long period and is one of the major carcinogens [26, 30]. Cancers that are strongly related to smoking might therefore also be related to air pollution that contains PAHs. The concentrations of PAHs in outdoor air pollution are much lower than that in tobacco smoking, but large populations are exposed for a long time. Therefore, even a marginal increase at air pollution level could lead to higher cancer risks at the population level.

Other possible underlying mechanisms of the deleterious effect of air pollution or particulate matter on cancer are listed as follows. Air pollution was considered as an oxidative stressor that led to inflammation and cell death [31]. DNA damage such as PAH-DNA adducts detected in human esophagus samples from Linxian, China [32] and DNA methylation observed in mice breathing high-efficiency air particulate filtered ambient air [33] appeared to support the causal role of PAHs or particulate matter exposure in epigenetic perspective. Exposure to particulate matter might also be related to histone modifications [34] and either up- or down-regulated miRNA changes [35].

This research will extend the analysis of the geographical variations in esophageal cancer. Air pollution is certainly not the only reason that increases the risk of esophageal cancer among people. It might only contribute slightly when compared with tobacco smoking and other risk factors. Yet large populations are exposed for a long time. Therefore, even a marginal increase at air pollution level might lead to higher risks at the population level. Further studies will be required to prove the relationship between air pollution and esophageal cancer from different aspects and on a large sample scale.

## MATERIALS AND METHODS

### Study population and data collection

The Shandong Center for Disease Control and Prevention (SDCDC) cancer registry is the premier source for cancer statistics in Shandong Province. We obtained city-level age-standardized esophageal cancer mortality rates of all 17 cities in Shandong Province in 2015 from SDCDC. As a control, we also obtained city-level age-standardized mortality rates of all cancers combined, diabetes mellitus and ischemic heart disease in 2015 to demonstrate the specific effect of air pollution on esophageal cancer. We then collected detailed demographical, clinical and pathological information of the eligible patients in Qilu Hospital to estimate the effect of atmospheric PM_10_ on clinicopathological features of ESCC. We recruited all eligible patients from Qilu Hospital who underwent radical resection of esophageal cancer (n=1,255). The detailed inclusion and exclusion criteria are listed in Figure 3. This study was approved by Qilu Hospital of Shandong University's Ethics Review Committee. All participants have provided their written informed consent to participate in this study.

### Exposure assessment

We obtained air pollution data of PM_10_, SO_2_ and NO_2_ from Shandong Environmental Protection Bureau (SDEPB). These three types of data were annual mean monitoring data achieved from official air pollution monitoring stations. Totally, there were 144 available stations locating in all 17 cities during the year 2009 to 2014. We then calculated city-level mean pollution data to represent the pollution level of a certain city. The National Standard of China, Ambient Air Quality Standard (GB3095-1996) stipulated the measurement methods used for air quality assessment [36]. Gravimetric method, chemiluminescence method and ultraviolet fluorescence method were used to measure PM_10_, NO_2_ and SO_2_, respectively. The annual mean concentrations of pollutants were calculated based on at least 60 equally distributed daily values for PM_10_, and 144 equally distributed daily values for NO_2_ and SO_2_. Data were recorded as integral numbers in each station. City-level smoking rates in the year 2013 were gained from SDCDC. There are 17 cities in Shandong Province. However, two cities lacked the smoking registry data. So we only gained smoking rates of 15 cities.

### Statistical analysis

Ecologic analyses were made to examine the associations between air pollution and esophageal cancer mortality. The city-level mean concentrations of PM_10_, SO_2_, NO_2_ were plotted against the age-standardized mortality rates of esophageal cancer in 2015, respectively. Scatter plots were made (Figure 2) and correlation analyses were then processed. Normality test was made for each variable. Pearson correlation analysis was used when both variables followed normal distributions. Spearman rank correlation analysis was used when either variable was non-normally distributed. The results of *r/r_s_* values and *p* values of correlation analyses between air pollution and esophageal cancer mortality rates were listed in Table 1. *p* values less than 0.05 were considered as statistically significant. To demonstrate the specific effect of air pollution on esophageal cancer, we also made scatter plots and correlation analyses to estimate the associations between air pollution and mortality rates of all cancers combined, diabetes mellitus and ischemic heart disease. The results were presented in Supplementary Figure 1 and Supplementary Table 3. Multiple linear regression analyses of esophageal cancer mortality rates were run with the combination of mean concentrations of PM_10_, SO_2_, NO_2_ and city-level smoking rates. Smoking was taken into consideration because it was a risk factor that increased esophageal cancer mortality [37]. Esophageal cancer mortality rates were considered as dependent variable. Collinearity diagnostics was made on each model to test if there was collinearity between the independent variables. Statistics significant models were listed in Table 2.

To further estimate the effect of atmospheric PM_10_ on clinicopathological features of ESCC, we compared detailed medical information between different pollution level groups. All the eligible patients were divided into two groups. Each participant was assigned to either the high PM_10_ level group or the low PM_10_ level group according to the ambient PM_10_ level of their residence. PM_10_ levels were classified according to the National Standard of China, Ambient Air Quality Standard (GB3095-1996) [36]. Patients who resided in cities with Class 1, Class 2 or Class 3 PM_10_ levels were classified in the low PM_10_ level group. Patients who resided in cities with higher than Class 3 PM_10_ level were classified in the high PM_10_ level group. We introduced the most relevant confounder, cigarette-smoking status, as an adjustment to minimize the bias. Smoking shares some properties with air pollution [1, 26]. Besides, smoking can increase the incidence and mortality of esophageal cancer [11, 37] as well as affect the clinicopathological features of esophageal cancer (Supplementary Table 4). Cigarette-smoking status was used as a standard to categorize patients into three subgroups, current smokers, former smokers and never smokers. Clinicopathological features of both groups, including demographics (gender, age at diagnosis), clinical and pathological characteristics (tumor location, primary tumor invasion, regional lymph nodes metastasis, histological grade, stage, lymph-vascular invasion and tumor size) were compared. Cochran-Mantel-Haensel chi-square test was used for categorical variables. Analysis of covariance was used for numerical variables. *p* values less than 0.05 were considered statistically significant. All statistical analyses were conducted using SPSS 20.0 software (SPSS Inc. Chicago, Illinois, USA).

## SUPPLEMENTARY MATERIALS FIGURES AND TABLES



## Abbreviations

ESCC: esophageal squamous cell carcinoma
PM_10_: particulate matter less than 10 μm in aerodynamic diameter
PM_2.5_: particulate matter less than 2.5 μm in aerodynamic diameter
IARC: International Agency for Research on Cancer
SDCDC: Shandong Center for Disease Control and Prevention
SDEPB: Shandong Environmental Protection Bureau
PAHs: polycyclic aromatic hydrocarbons.
