# Supplementary material for: The effects of air pollution on mortality and clinicopathological features of esophageal cancer
Source: Oncotarget. 2017 Apr 20;8(35):58563–76. doi: 10.18632/oncotarget.17266 (PMC5601675; doi:10.18632/oncotarget.17266)
Supplement: Supplementary file 1 [file oncotarget-08-58563-s001.pdf]

The effects of air pollution on mortality and clinicopathological features of esophageal cancer

SUPPLEMENTARY MATERIALS

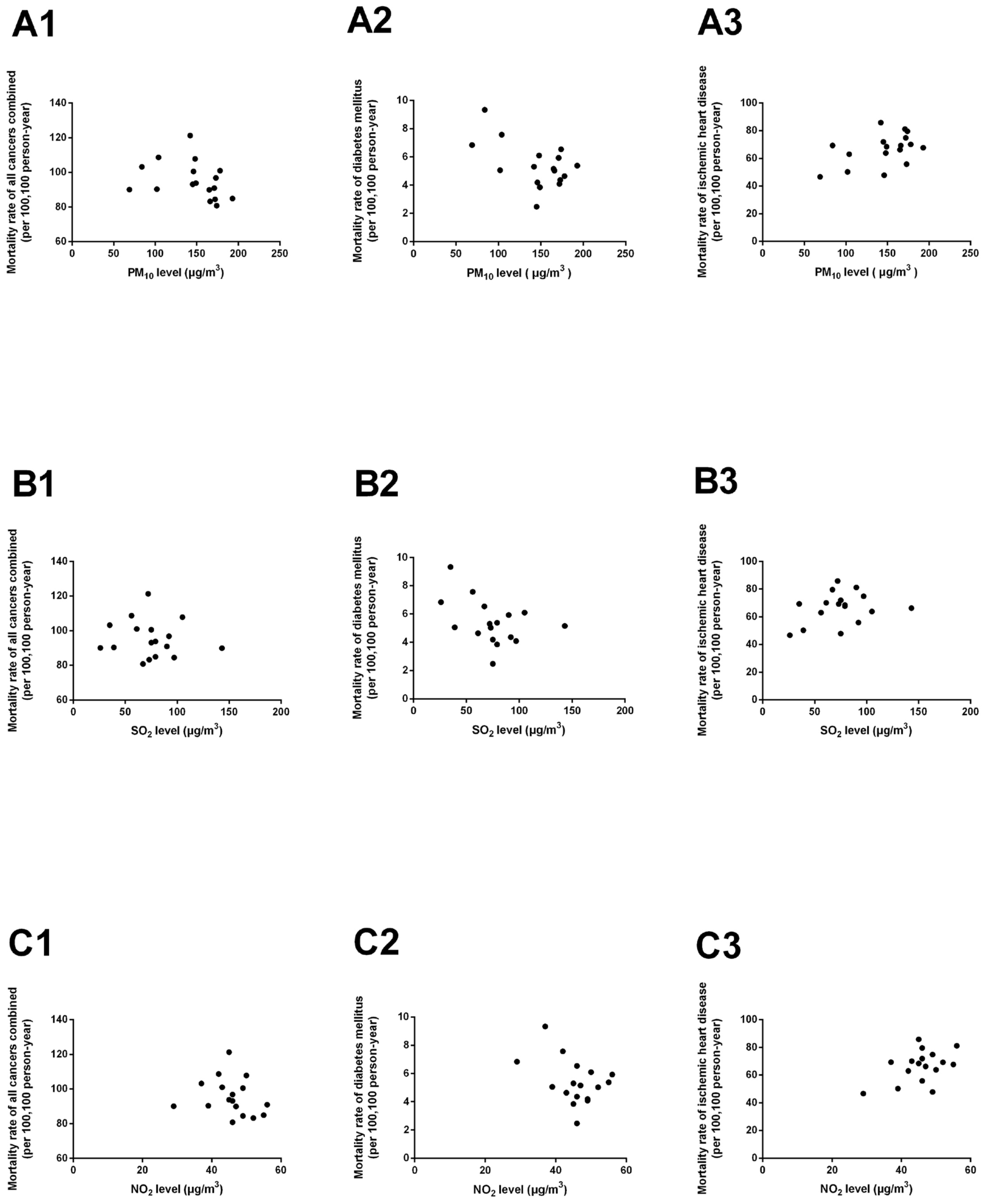

Supplementary Figure 1: Scatter plots of air pollution concentrations against mortality rates of all cancers combined, diabetes mellitus and ischemic heart disease in Shandong Province. The city-level mean concentrations of PM_{10}, SO_2, NO_2 were plotted against the age-standardized city-level mortality rates of all cancers combined, diabetes mellitus and ischemic heart disease in 2015, respectively.

Supplementary Table 1: Mean concentrations of PM_{10}, SO_2 and NO_2 during 2009-2014 of all 17 cities in Shandong Province

City	PM_{10} concentration (μg/m^3)		SO_2 concentration (μg/m^3)		NO_2 concentration (μg/m^3)	
	Mean	Std. deviation	Mean	Std. deviation	Mean	Std. deviation
Jinan	171.00	14.94	89.67	12.47	55.67	5.28
Qingdao	104.33	15.16	55.50	14.60	42.00	4.86
Zibo	165.33	16.34	143.17	26.53	47.00	14.23
Zaozhuang	172.83	12.92	92.17	7.36	46.00	4.73
Dongying	146.33	17.50	74.83	9.11	49.17	3.60
Yantai	84.33	9.11	34.67	5.92	36.67	1.51
Weifang	149.33	16.57	79.33	16.11	44.67	1.21
Tai’an	142.33	15.88	71.67	17.57	44.50	3.33
Weihai	69.00	4.05	26.17	3.06	28.83	4.49
Laiwu	148.17	11.46	105.00	19.82	49.83	7.33
Linyi	193.33	21.94	79.33	20.90	54.67	3.39
Dezhou	165.67	15.81	72.67	14.40	52.33	5.72
Liaocheng	174.33	30.36	67.33	8.80	46.00	5.87
Binzhou	144.50	15.67	74.83	8.26	45.50	3.51
Heze	177.67	17.18	60.67	8.09	42.50	4.14
Jining	171.83	9.41	96.67	17.80	49.33	3.67
Rizhao	102.00	13.52	39.17	4.36	39.00	3.03

Supplementary Table 2: Multiple linear regression modeling of factors associated with esophageal cancer mortality (all models)

Model		*β* (95% CI)	Std. error	*t*	*R2*	*F*	*p*
1	Constant	4.56 (-2.69, 11.81)	3.29	1.39	0.62	9.14	**<0.01**
	Smoking	-0.25 (-0.53, 0.04)	0.13	-1.89			
	PM_{10}	**0.05 (0.03, 0.08)**	0.01	4.18			
2	Constant	-0.33 (-11.36, 10.71)	5.01	-0.07	0.45	4.45	0.04
	Smoking	-0.15 (-0.49, 0.19)	0.15	-0.97			
	NO_2	**0.23 (0.05, 0.40)**	0.08	2.89			
3	Constant	6.84 (-4.28, 17.95)	5.05	1.35	0.15	0.96	0.41
	Smoking	-0.11 (-0.53, 0.31)	0.19	-0.57			
	SO_2	0.03 (-0.02, 0.08)	0.02	1.25			
4	Constant	4.29 (-6.46, 15.03)	4.82	0.89	0.63	5.55	0.02
	Smoking	-0.25 (-0.56, 0.07)	0.14	-1.75			
	PM_{10}	0.05 (-0.001, 0.11)	0.02	2.18			
	NO_2	0.01 (-0.26, 0.28)	0.12	0.08			
5	Constant	5.44 (-1.90, 12.78)	3.29	1.65	0.67	6.9	**0.01**
	Smoking	-0.29 (-0.58, 0.01)	0.13	-2.18			
	PM_{10}	**0.07 (0.03, 0.10)**	0.02	4.02			
	SO_2	-0.03 (-0.07, 0.02)	0.02	-1.24			
6	Constant	-0.64 (-12.39, 11.12)	5.28	-0.12	0.46	2.79	0.10
	Smoking	-0.16 (-0.52, 0.20)	0.16	-0.97			
	SO_2	-0.01 (-0.07, 0.05)	0.03	-0.40			
	NO_2	0.26 (0.02, 0.49)	0.11	2.38			
7	Constant	4.29 (-6.35, 14.93)	4.70	0.91	0.68	4.75	0.02
	Smoking	-0.28 (-0.59, 0.04)	0.14	-2.00			
	SO_2	-0.03 (-0.08, 0.02)	0.02	-1.23			
	NO_2	0.04 (-0.23, 0.32)	0.12	0.36			
	PM_{10}	**0.06 (0.01, 0.12)**	0.02	2.50			

Multiple linear regression analyses were run with the combination of mean concentrations of PM_{10}, SO_2, NO_2 and city-level smoking rates. Esophageal cancer mortality rates were considered as dependent variable. Mean concentrations of PM_{10}, SO_2, NO_2 and city-level smoking rates were considered as independent variables. Collinearity diagnostics was made on each model and there was no collinearity between the independent variables. All multiple linear regression analyses models were listed. Bonferroni correction was applied. *p* values less than 0.05/n were considered statistically significant, where n was the number of independent variables.

Supplementary Table 3: Correlation analyses between air pollution and mortality rates of all cancers combined, diabetes mellitus and ischemic heart disease

Correlation analysis	r/r_s	p
PM_{10} & mortality rates of all cancers combined	-0.42[a]	0.09[a]
PM_{10} & diabetes mellitus mortality rates	-0.30[a]	0.25[a]
PM_{10} & ischemic heart disease mortality rates	0.35[a]	0.17[a]
SO_2 & mortality rates of all cancers combined	-0.10	0.70
SO_2 & diabetes mellitus mortality rates	-0.42	0.10
SO_2 & ischemic heart disease mortality rates	0.28	0.28
NO_2 & mortality rates of all cancers combined	-0.21	0.41
NO_2 & diabetes mellitus mortality rates	-0.38	0.13
NO_2 & ischemic heart disease mortality rates	0.44	0.08

Correlation analyses were processed between city-level mean concentrations of PM_{10}, SO_2, NO_2 and city-level age-standardized mortality rates of all cancers combined, diabetes mellitus and ischemic heart disease in 2015, respectively. The results of r/r_s values and p values were listed. Normality test was made for each variable. Pearson correlation analysis was used when both variables followed normal distributions. Spearman rank correlation analysis was used when either variable was non-normally distributed. p values less than 0.05 were considered statistically significant.

[a] Results gained using Spearman rank correlation analysis

Supplementary Table 4: Clinicopathological features between different cigarette-smoking status groups

Variables	Cigarette -smoking status			χ^2/F	*p*
	Never (523)	Current (577)	Former (155)		
Tumor location				12.02	0.06
Cervical	21 (1.67%)	8 (0.63%)	2 (0.16%)		
Upper thoracic	37 (2.95%)	28 (2.23%)	8 (0.64%)		
Middle thoracic	314 (25.02%)	363 (28.92%)	100 (7.97%)		
Lower thoracic	151 (12.03%)	177 (14.10%)	45 (3.59%)		
Primary tumor invasion				24.75	**<0.01**
Tis	16 (1.27%)	11 (0.88%)	3 (0.24%)		
T1	58 (4.62%)	45 (3.59%)	13 (1.04%)		
T2	169 (13.47%)	139 (11.08%)	40 (3.19%)		
T3	217 (17.29%)	291 (23.19%)	66 (5.26%)		
T4	62 (4.94%)	88 (7.01%)	33 (2.63%)		
Regional lymph nodes				15.15	**0.02**
N0	318 (25.34%)	292 (23.27%)	85 (6.77%)		
N1	119 (9.48%)	149 (11.87%)	42 (3.35%)		
N2	59 (4.70%)	101 (8.05%)	22 (1.75%)		
N3	27 (2.15%)	35 (2.79%)	6 (0.48%)		
Histological grade				2.19	0.70
G1	90 (7.17%)	96 (7.65%)	26 (2.07%)		
G2	225 (17.93%)	228 (18.17%)	61 (4.86%)		
G3	208 (16.57%)	253 (20.16%)	68 (5.42%)		
Stage				21.20	**<0.01**
0	15 (1.20%)	11 (0.88%)	3 (0.24%)		
I	59 (4.70%)	49 (3.90%)	13 (1.04%)		
II	262 (20.88%)	239 (19.04%)	63 (5.02%)		
III	184 (14.66%)	274 (21.83%)	76 (6.06%)		
IV	2 (0.16%)	1 (0.08%)	0		
Lymph-vascular invasion				4.22	0.12
No	508 (40.48%)	548 (43.67%)	151 (12.03%)		
Yes	15 (1.20%)	29 (2.31%)	4 (0.32%)		
Tumor size (cm)	3.65 ± 1.69	3.99 ± 1.71	4.13 ± 2.14	7.16	**<0.01**
Diagnostic age	61.00 ± 8.15	59.68 ± 7.83	62.44 ± 7.72	8.63	**<0.01**
Gender				359.68	**<0.01**
Male	299 (23.82%)	572 (45.58%)	154 (12.27%)		
Female	224 (17.85%)	5 (0.40%)	1 (0.08%)		
Comorbidities				17.03	**<0.01**
No	385 (30.68%)	451 (35.94%)	96 (7.65%)		
Yes	138 (11.00%)	126 (10.04%)	59 (4.70%)		

Whole percentages were used. *p* values less than 0.05 were considered statistically significant.
